# Open-source, MRI-compatible grip force sensor for dynamic muscle imaging

**DOI:** 10.1007/s10334-025-01282-y

**Published:** 2025-07-25

**Authors:** Sabine Räuber, Regina Schlaeger, Marta Brigid Maggioni, Francesco Santini

**Affiliations:** 1 Department of Biomedical Engineering, Basel Muscle MRI (BAMM), University of Basel, Basel, Switzerland; 2 Department of Radiology and Nuclear Medicine, University Hospital of Basel, Basel, Switzerland; 3 Department of Clinical Research, University of Basel, Basel, Switzerland; 4 Neurologic Clinic and Policlinic, University Hospital Basel, Basel, Switzerland

**Keywords:** Muscles, Electric stimulation, MRI, 3D printing

## Abstract

**Objective:**

Dynamic MRI synchronised with neuromuscular electrical stimulation (NMES) offers a reproducible method for assessing muscle activity but requires MRI-compatible force sensors to correlate quantitative muscle dynamics parameters with muscle force output. Most available sensors are expensive, rely on non-free software or are MR-incompatible This work presents an open-source, low-cost, MR-compatible grip force sensor as a viable alternative to commercial devices.

**Materials and Methods:**

Phantom measurements were performed with and without the sensor at a 3T MRI to assess the MRI compatibility and its impact on image quality, field homogeneity and signal-to-noise ratio (SNR). Furthermore, the force sensor was integrated into a dynamic MRI setup with NMES and applied in vivo to four subjects.

**Results:**

The force sensor demonstrated good compatibility with a 3 T MRI scanner, exhibiting minimal SNR reduction and minimal increase in B_0_ inhomogeneities in phantom measurements. During dynamic MRI with NMES, a 2D in-plane phase-contrast MRI sequence successfully retrieved the muscle’s velocity field, proving effective for dynamic MRI applications, while preserving image quality.

**Discussion:**

The design of the force sensor, building instructions and software are publicly released as open source. This allows the proposed sensor to be adapted in multiple applications where grip force needs to be recorded in an MR scanner.

**Supplementary Information:**

The online version contains supplementary material available at 10.1007/s10334-025-01282-y.

## Introduction

Myotonic dystrophy type 1 is a genetic, multisystemic disorder affecting skeletal and smooth muscle, heart, brain, eyes and other organs. Its global prevalence ranges from 0.37 to 36.29 per 100,000 individuals, with a pooled estimated prevalence of 9.27 cases per 100,000 [1], making it one of the most common forms of muscular dystrophy in adults. This condition manifests with various clinical symptoms, including progressive muscle weakness, myotonia, early cataracts, cardiac conduction abnormalities, and other systemic dysfunctions. A distinctive clinical feature of myotonic dystrophy is grip myotonia [2], where patients experience difficulty releasing their grip due to prolonged muscle relaxation after contraction [3]. This characteristic symptom stems from alterations in muscle membrane excitability [4, 5], significantly impacts patients’ quality of life [6], and is currently difficult to quantitate on clinical grounds alone. Dynamic MRI of muscle contraction, either during voluntary motion or evoked by neuromuscular electrical stimulation (NMES), allows calculating velocity, strain and strain rate maps [7–10], providing valuable insights into muscle functional performance, such as alterations in velocity, deformation, and contractile properties, in both the healthy muscle [7, 8] as well as in neuromuscular diseases [9, 11]. This approach could be particularly helpful in refining our understanding of the phenomenon of myotonia in patients with myotonic dystrophy, in quantifying its extent and potentially in monitoring the effects of symptomatic as well as disease-modifying interventions in the future.

Dynamic MRI synchronised with NMES, when combined with physical force measurements, offers a reproducible method for assessing muscle activity. This approach allows for the normalisation of the strain to the maximum applied force [11, 12]. However, this method requires MRI-compatible force sensors that are both safe and do not cause image artefacts. Grip force measurement is particularly relevant for characterising grip myotonia using dynamic MRI.

Multiple commercially available devices have already been developed to measure grip force in an MRI environment [13–16] and are primarily utilised in the context of functional MRI. However, commercial devices often cost upwards of 2000 USD and require proprietary add-on software. Furthermore, such commercial devices are based on optical fibre sensors, which are non-magnetic and non-conductive, making them ideal candidates for use in an MRI scanner, where the magnetic fields that are used for the acquisition (either constant or time-varying) may cause mutual interference with the sensor’s hardware. However, optical fibre sensors are, depending on the specific type, susceptible to intensity fluctuation either due to light source instability, fibre bending or fibre mismanagement. They are also expensive and subject to repetitive recalibration over time [17]. Some optical grip force sensors have also been developed in academic settings [17–19] for research purposes. However, the building instructions of the published designs are not open source.

This lack of accessibility hinders their replication and applicability for force measurements in dynamic muscle MRI. This work presents an open-source, low-cost, MR-compatible force sensor to measure grip force during dynamic MR acquisition. This sensor is based on a previously released open-source foot pedal design [20].

The device allows for accurate grip force measurement and was built with non-ferromagnetic materials (brass screws, plastic casing, and aluminium load cells) to prevent magnetic interference. Cables and other components are shielded to reduce electromagnetic interference from gradients and radiofrequency (RF) pulses. The total material costs are low compared to commercial solutions, as the sensor has standard off-the-shelf materials. Building instructions and a material list are publicly available under the Creative Commons -Attribution (CC-BY) version 4.0 licence at https://github.com/BAMMri/Open-Grip-Force [21].

## Methods

### Device design and setup

**Fig. 1:**
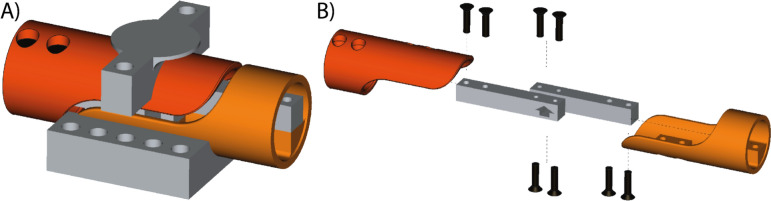
**A** Scheme of the grip force sensor with load cells inside the 3D-printed casing (orange) connected in parallel for accurate force measurement. The calibration module is indicated in grey. **B** An exploded view of the force sensor components, where the load cells are static objects, ensuring their position remains fixed to one another and serving as a reference point for the exploded assembly casing

A handheld device was designed using FreeCAD, an open-source parametric 3D computer-aided design (CAD) modeller (version 0.21.2), and subsequently 3D printed with a commercial fused-deposition-modelling 3D printer (A1, Bambu lab, Shenzhen, China) to house the active force measurement elements (see Fig. 1), which deform upon grip-induced force (simplified schematic force diagram in the supplementary information Fig. S1). A schematic sketch of the electronic components can be found in Fig. S2. The comprehensive printing settings and the detailed FreeCAD model of the device are available at https://github.com/BAMMri/Open-Grip-Force [21].

The 3D enclosure incorporates two Wheatstone-bridge aluminium beam load cells (50 kg/490 N each) to accommodate the typical maximal grip force range of myotonic dystrophy patients (3–50 kg/30–490 N) [22] and healthy subjects (up to 83 kg/814 N) [23]. The load cells are secured to the 3D-printed casing with brass screws, and electrically connected in a parallel configuration to effectively act as a single 980 N load cell. These cells interface with an HX711 amplifier and an analogue-to-digital converter (ADC), delivering a temporal resolution of 100 ms, which can be tuned to 10 ms (selectable on the amplifier board).

The ADC is operated and read by an Arduino Uno [24] microcontroller, placed outside the scanner room, which continually collects force data and transmits it via a USB connection to a PC. An in-house developed Python program [20] (Python version 3.13.0) receives these data and outputs it in a readable format, storing force values in either Newtons or Kilograms in a text file. To mitigate bidirectional electromagnetic interference from and to the scanner, the sensors are connected to the ADC through shielded Ethernet cables, reinforced by copper braiding at solder junctions. Furthermore, the length of the Ethernet cable within the scanner bore, and consequently the potential RF exposure of the cable, is minimised by feeding the wires from behind the scanner for a superman (head-first) MRI.

**Fig. 2:**
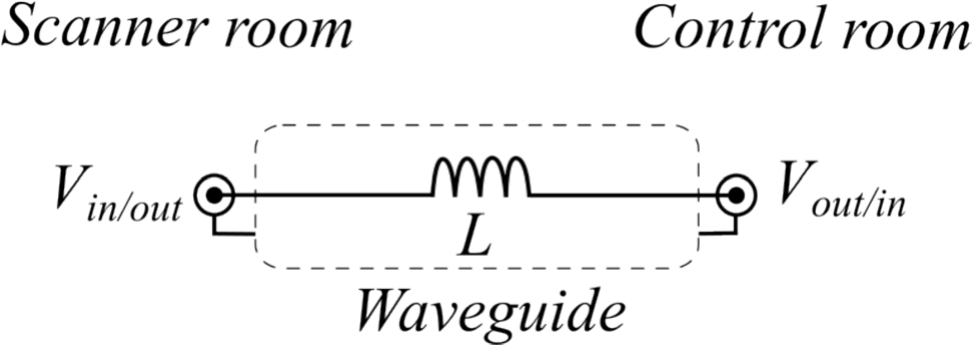
Electrical schematics of the low-pass filter for each cable. The filter receives inputs from the force sensor, NMES and trigger inside/outside the scanner room and outputs these signals to/from the Arduino microcontroller. The filter is located inside the waveguide in the wall of the MR scanner room to protect the microcontroller from resets due to RF interference

The sensor’s electrical connections to the HX711 board (four cables connected to the four endpoints of the Wheatstone bridge) pass through a custom-designed low-pass filter built with 100-μH inductors, which safeguards the Arduino microcontroller from gradient- and RF-induced voltages while minimising electromagnetic noise in the MRI image. This common-mode filter has been specifically designed to fit within the waveguide tube of the scanner room with the cable shielding grounded through its connection to the MRI system’s RF shielding (see Fig. 2).

The filter also included two additional lines for the delivery of an optional NMES stimulus. This extension of the grip force setup included an NMES device (EM 49, Beurer GmbH, Germany) positioned outside the scanner room. One channel of the device connects through the low-pass filter with two rectangular gel electrodes (5.1 × 8.9 cm^2^ from TensUnits.com, USA) to the volunteer’s forearm above the finger flexor muscles to evoke a hand grip, while the other channel is linked to the microcontroller and serves as a trigger for MRI acquisition. The NMES setup has undergone safety testing for RF-induced heating [25] and has been utilised during MRI imaging previously, as reported in Ref. [8]. For additional safety, direct contact between subjects and cables is avoided.

### Calibration measurements

A calibration module was also 3D printed to facilitate the calculation of the scale factor and assess the accuracy of the grip force sensor (see Fig. 1, 3D model available in the same repository). This device securely sandwiches the sensor between two plates that are fastened with screws, allowing for the systematic placement of multiple known weights, ranging from 0.1 to 100 kg, on the sensor outside the scanner room. As each weight is applied, the sensor logs the corresponding measured force, which allows for determining a scale factor that correlates the sensor’s output with the reference weight. As in

$${\text{Fscale}}_{\text{new}}= \frac{{\text{Fscale}}_{\text{old}} \times {\text{weight}}_{\text{measured}} }{{\text{weight}}_{\text{reference}}}$$

To verify that the calibration was not influenced by the MRI scanner, we performed an additional calibration using ten different weights inside the MRI scanner while a GRE sequence was running. Due to the limited availability of MRI-compatible weights, the calibration was restricted to a maximum weight of 10 kg.

### MRI measurements

The compatibility of the device with the MR scanner was evaluated both in a cylindrical water phantom and in-vivo measurements in a 3 T whole-body MRI scanner (MAGNETOM Prisma, Siemens Healthineers, Erlangen, Germany) equipped with a gradient system capable of 80 mT/m amplitude and 200 T/m/s slew rate, an 18-channel flex coil was used for signal acquisition.

### Validation in phantom

For the phantom-based measurements, a gradient-recalled echo (GRE) sequence was employed for the SNR calculation, utilising the following parameters: flip angle = 25°, echo time (TE) = 3.84 ms, and a spatial resolution of 1.0 × 1.0 × 2.0 mm3. In addition, the reconstructed phase images provided by the scanner were used in a dual echo acquisition (TE1/TE2 = 3.84/9.09 ms) to investigate potential effects of the device on field homogeneity (B_0_). The signal-to-noise ratio (SNR) was determined according to NEMA [26] standards, specifically using the second proposed method for SNR calculations. In this approach, the noise reference is established from an acquisition with no MRI signal. Ten separate acquisitions of both the signal and pure noise were conducted to perform a t-test. For qualitative analysis, additional RF noise spectra were acquired using the Siemens RF noise service sequence. B_0_ mapping and RF noise spectra acquisitions were performed three times: a first baseline acquisition with no electronics and no force sensor, a second experiment with the electronics installed in the MRI room and connected through the filter to the control room, and finally with the electronics and the force sensor active and positioned next to the phantom cap (outside the coil). During this last scenario, the calibration module was also attached to the device, and a force of 100 N was applied. SNR measurements were performed twice, once with the force sensor next to the phantom and once without.

### MRI measurements (validation in vivo)

An anatomical T1-weighted gradient-recalled echo (GRE) scan was acquired in vivo on one healthy subject (male, 43 years old) during isometric grip on the force sensor to assess potential device-induced artefacts. In this specific acquisition, the coil was entirely wrapped around the hand gripping the force sensor to investigate the extent of the generated artefacts (please note that this configuration differs from the proposed use case, in which the coil does not cover the force sensor). The scan parameters were as follows: flip angle = 25°, TE = 3.58 ms, resolution = 1 × 1  ×2 mm^3^, and bandwidth = 261 Hz/px.

In addition, a conventional 2D phase-contrast sequence (with in-plane flow sensitivity along the cranio-caudal axis of the subject) was acquired sagittally on n = 4 healthy subjects (two males, two females, ages ranging from 24 to 43 years old) while triggering with NMES. The sequence parameters included 58 temporal phases, a repetition time (TR) of 22.1 ms, TE of 7.28 ms, an in-plane resolution of 1.4 × 1.4 mm2, velocity encoding (venc) of 20 cm/s, and a total acquisition time (TA) of 2.30 min. The NMES protocol consisted of a train of bipolar rectangular pulses with 300 µs pulse duration at a frequency of 60 Hz, and a stimulation time of 0.78 s on and off. The total length of a stimulation cycle is therefore 1.56.

## Results

**Fig. 3:**
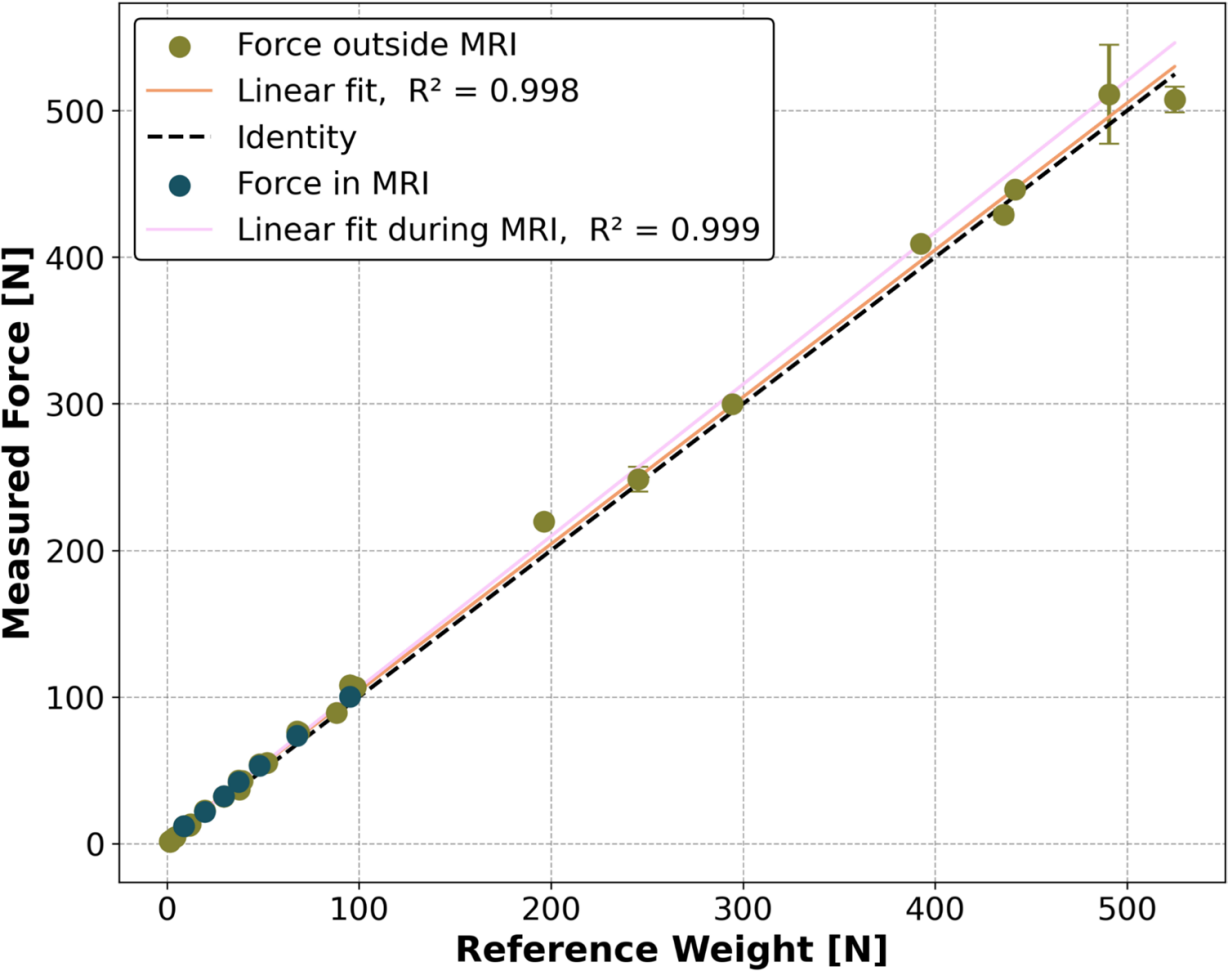
Grip force sensor sensitivity and linearity up to 588 N reference force outside of the MRI and 98 N inside the MRI. The green data points represent the mean values and standard deviation from three repeated weight placements on the sensor. The blue data points were acquired in the MRI scanner with a limited number of ten MRI safe weights, while a standard GRE was acquired. The orange line represents the linear regression outside of the MRI, the pink line within the MRI, while the black dashed line represents ideal linearity. The resulting linear regressions are in good agreement, demonstrating a reliable grip force measurement inside and outside the MRI

The force sensor demonstrated good linearity (up to 588 N) with an *R*2 value of 0.998 outside of the MRI and comparably good linearity (up to 98 N) with an *R*2 value of 0.999 inside the MRI, demonstrating a robust linear correlation between measured force and sensor output (Fig. 3).

**Fig. 4:**
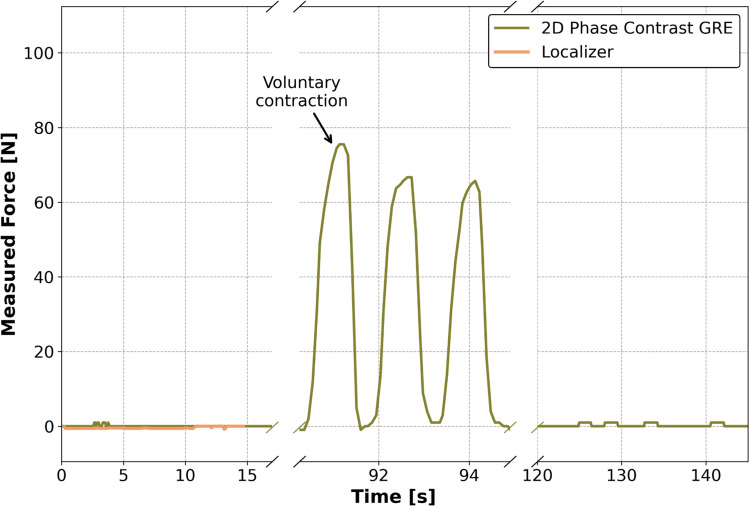
Recorded force plots during a localiser (0–15 s) and a 2D in-plane phase-contrast (0–155 s) MRI acquisition. The signal peaks due to voluntary grip force application (indicated by the arrow between 91 and 95 s), clearly distinguishable from background signal oscillations

Without the low-pass filter, electromagnetic interference caused the microcontroller to reset during MR acquisitions, while adding the filter enabled force values to be recorded throughout acquisitions. Minor signal oscillations of approximately 1 N in magnitude were observed qualitatively during the MRI acquisition of the localiser and a 2D phase-contrast GRE (Fig. 4). Nevertheless, the measured force during voluntary contractions of the grip force was of an order of magnitude greater and therefore clearly distinguishable from the background oscillations.

**Fig. 5:**
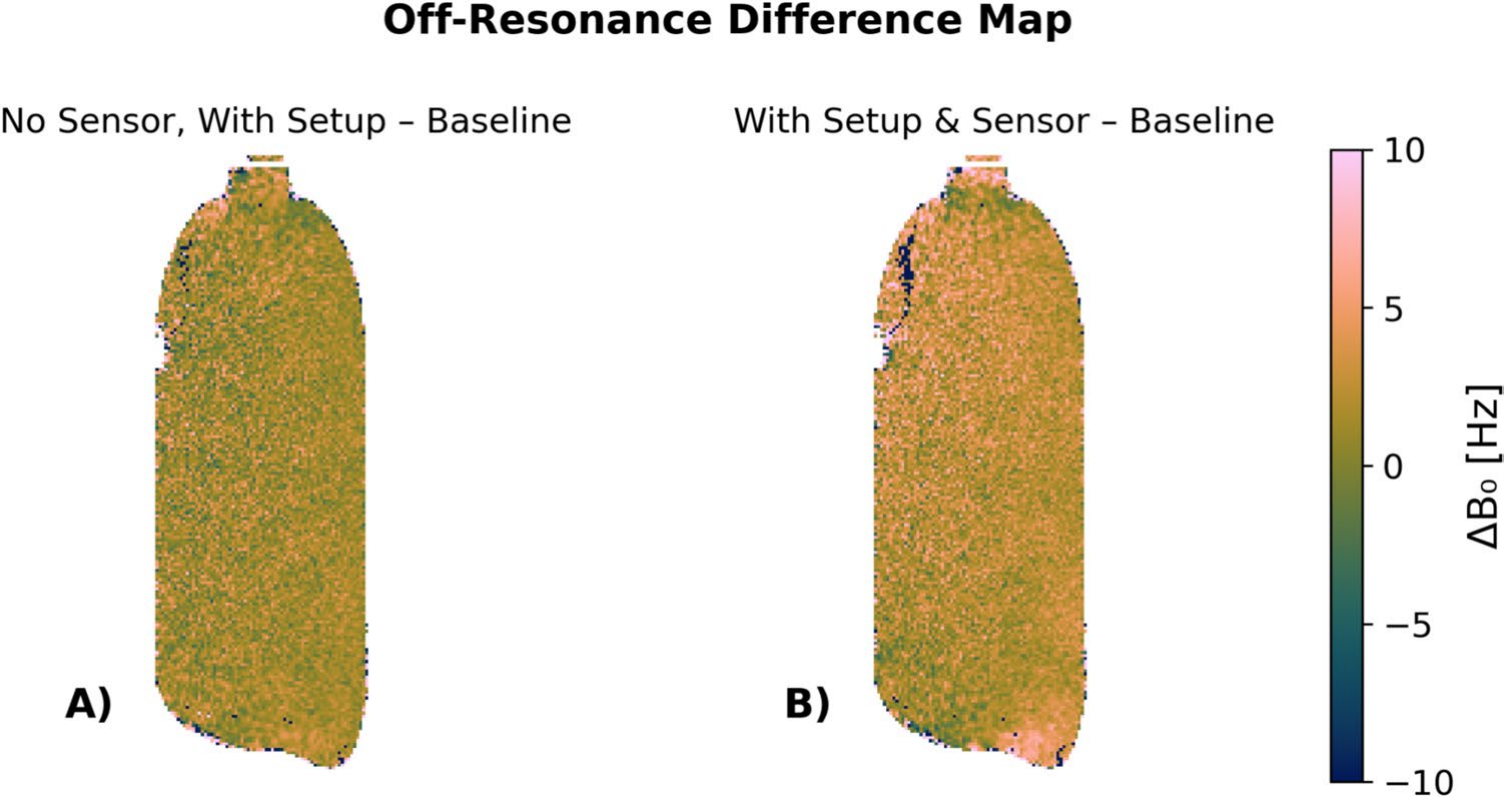
B_0_ difference map of a 0.5 L water phantom, showing the difference in the off-resonance field. The first map shows the deviation introduced by the setup alone [difference with the baseline where no setup is present (**A**)], while the second map highlights the effects of the active force sensor (**B**). On average, deviations of 1.0 ± 12 Hz and 1.3 ± 16 Hz indicate a minimal increase in field inhomogeneities and variability with each scenario

The B_0_ map deviations within the water phantom of 1.0 ± 12 Hz and 1.3 ± 16 Hz indicate a marginal increase in field inhomogeneities and variability for the scenarios with setup installed and setup installed and active force sensor within the MRI compared to the baseline without sensor and setup (see Fig. 5).

The measured SNR was reduced from 232 ± 5 to 223 ± 10 with the force sensor active during acquisition. The difference was statistically significant (*p* < 10^–4^), reflecting a 3.7% SNR drop with the connected device. Accordingly, the RF noise spectrum plot (Figure S3) shows comparable magnitudes of mean and maximum RF noise for both scenarios. However, when the force sensor is active during acquisition, a small peak appears in the noise spectrum around − 65 Hz from the proton resonance frequency.

**Fig. 6:**
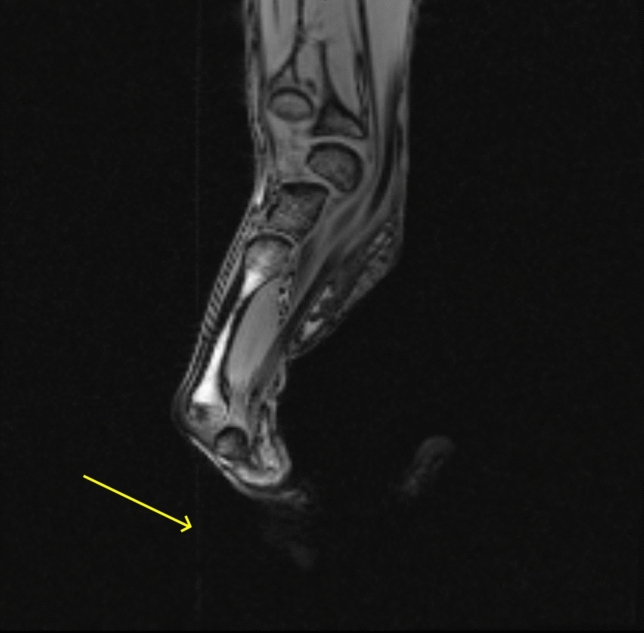
Anatomical T_1_-weighted GRE with an isometric grip of the force sensor. No artefacts are visible in the forearm. However, subtle (vertical) RF artefacts are visible near the force sensor, indicated by the arrow. Note that the grip force sensor is placed inside the coil, which is not the intended case of use

No susceptibility artefacts are visible in the forearm MRI, while subtle RF artefacts are perceptible near the force sensor (Fig. 6).

**Fig. 7:**
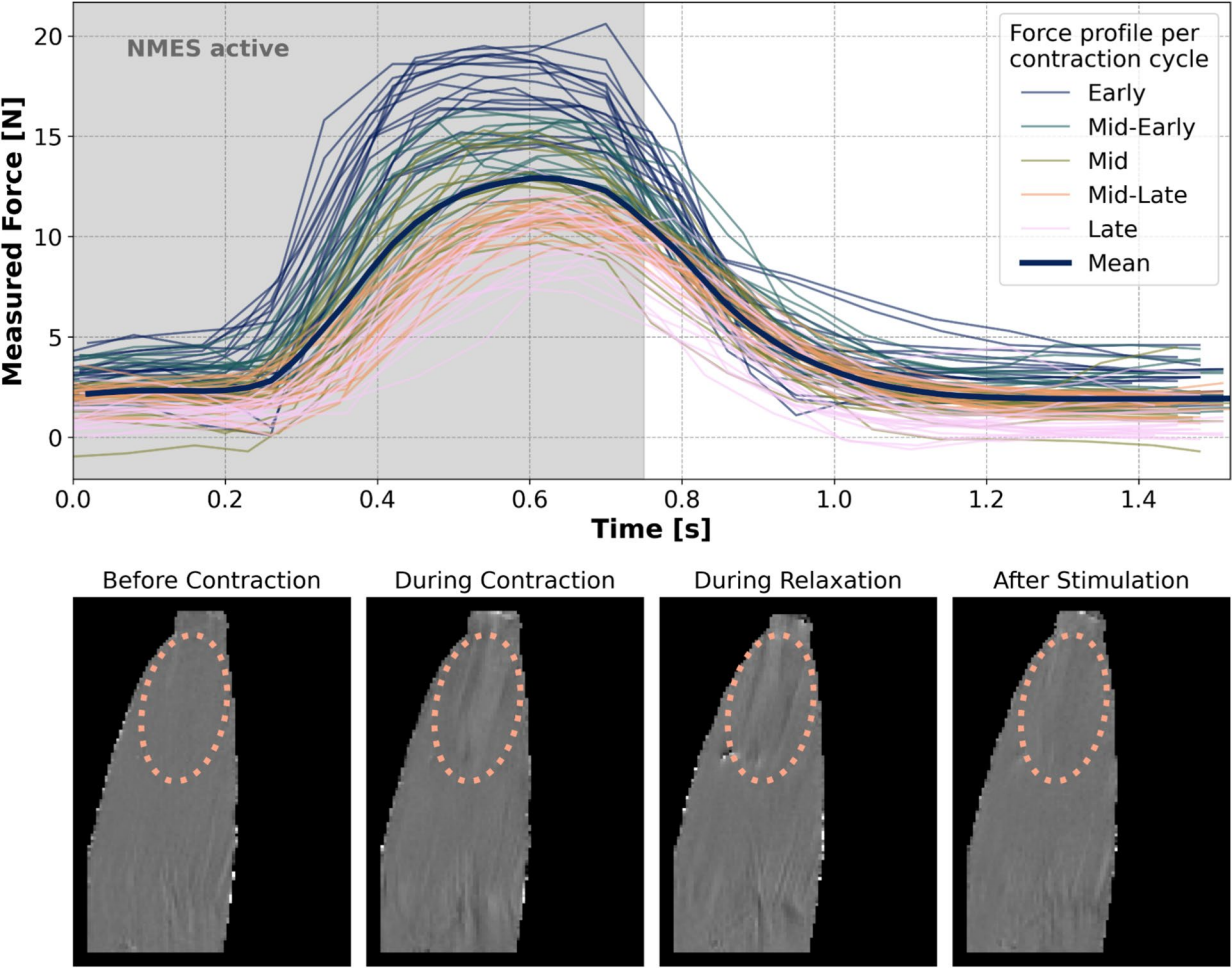
Measured force evoked by NMES stimulation during a phase-contrast acquisition per NMES cycle, upper panel. Each NMES cycle consists of 0.78 s on-time with 300 us pulses at 60 Hz (indicated by grey shading), followed by 0.78 s off-time. To make up for the averaging effects due to the temporal resolution of our device and trigger recordings, we subtracted a 0.05 s delay from our signal to improve the alignment of the force profiles per contraction. In total, 93 contractions were recorded, shown above with five different colours to distinguish early contractions at the beginning of the phase-contrast MRI acquisition from late contractions towards the end of the acquisition. The bottom panel shows velocity images from the phase-contrast MRI sequence showing the dynamic changes in the muscle: before contraction, during contraction, subsequent relaxation and final return to baseline after stimulation

The velocity and magnitude images of the NMES-evoked forearm contraction did not show any RF artefacts in any of the four in-vivo acquisitions. Velocity changes throughout the contraction cycle are qualitatively visible over time within a region of interest (Fig. 7). The corresponding measured grip force profile gradually decreases over time for the 93 NMES-evoked contraction during scanning of the 2D phase-contrast sequence. On average, the force profile increases above the initial baseline (0 N) after 0.2 s with NMES on. The grip force peaks at 0.6 s while NMES is still on. Around 1.2 s, the measured force output returns to the initial baseline of 0 N, while NMES is off.

## Discussion

In this paper, we presented an open-source implementation of a grip force sensor to be used in an MR environment. The device demonstrated robust functionality and a minimal penalty in terms of output image quality. In addition, the sensor can be integrated as part of an extended setup to consistently measure evoked muscle contractions induced by neuromuscular electrical stimulation throughout each contraction cycle. The velocity was measured through synchronised acquisition with 2D in-plane phase-contrast MRI sequences. This work serves as proof of functionality towards subsequent applied studies, where strain and strain rate maps can be calculated from the velocity fields and normalised to the applied grip force, as done in previous studies on leg muscles [11, 12].

This sensor is built with commercially available materials that are not specifically designed for operation in an MR environment, and, indeed, variations in B_0_ homogeneity and a reduction in SNR during sensor activity were observed. However, the impact of both effects was minimal. In particular, B_0_ variations were small, even compared to B_0_ inhomogeneities induced by differences in susceptibility of anatomical structures in various regions of the body [27, 28]. As expected, the sensor’s presence reduced SNR by increasing noise levels within the scanner. A 3.7% reduction is considered an acceptable limitation for the grip force measurements in this work. However, this reduction may pose challenges for applications with intrinsically lower SNR, such as imaging and spectroscopy of other nuclei.

No evident image artefacts are observed, even when deliberate efforts are made to produce them by positioning the force sensor within the coil, a setup outside normal use cases. Only a subtle RF artefact becomes visible upon careful windowing in the GRE image.

The low-pass filter installed in the waveguide allowed for force data acquisition during a sequence with strong gradients that could induce eddy currents and RF noise. Previous studies that did not use this filter [7–9, 12, 20, 29] achieved force recording by introducing a pause after the scanner’s self-calibration routine [20] and before initiating the measurement. The filter, proposed in this work, streamlines this process to ensure reliable force recording by the microcontroller.

For the force sensor to be suitable for dynamic muscle MRI studies, it must exhibit good linearity across a broad force range. The literature shows that healthy subjects have a maximum voluntary grip force of up to 83 kg/814 N [23] with a mean of around 56 kg/549 N [22], varying widely with age and gender while myotonic dystrophy patients exhibit a maximum voluntary grip force of up to 50 kg/490 N [22]. NMES typically evokes up to 20% of maximum voluntary contraction [35]. The force sensor, implemented in this work, has excellent linearity and can therefore adequately measure both healthy subjects and subjects with underlying disorders with or without NMES.

However, the grip force sensor is direction-dependent, meaning it acquires the force on one axis, perpendicular to the load cells. Therefore, variations in the measured force can occur due to changes in the device's orientation in the subject’s hand relative to the force axis. In the literature, efforts have been made to develop several multi-axis grip force sensors independent of the device orientation [30]. Unfortunately, these setups employ magnetic materials or lack open-source building instructions. Thus, we currently rely on the accurate positioning of the device in the hand of the subject to have comparable directions of force application across experiments. However, to ensure alignment with the axis of force and to improve reproducibility between and within subjects, ergonomic grip features can be incorporated into the casing. These features could include finger grooves and an asymmetric shape of the device’s casing to ensure correct hand positioning relative to the force-measuring axis of the device.

During dynamic MR acquisition, it is crucial to ensure reliable force measurements by minimising external sources of motion and RF interference. On occasion, we have observed that free wrist jerks of some subjects in response to NMES have led to the measurement of negative force values, which were eliminated with adequate fixation of the wrist and forearm within the coil using vacuum suction paddings. It is plausible that the motion of the load cells within the magnetic field leads to changes in magnetic flux, inducing currents that result in transient voltage affecting the measured force. Therefore, it is mandatory to restrict forearm movement during dynamic MR acquisition for reliable force measurements. Furthermore, particular care must be taken to reduce the Ethernet cable length within the scanner bore to prevent RF power-dependent offsets, which were observed in the force sensor recordings if proper cable management was not applied.

The time delay between the activation of the NMES impulse and the recorded maximum force plateau in Fig. 7 is due to a well-documented physiological phenomenon that is referred to as electromechanical delay, i.e. a time lag between electrical muscle excitation and subsequent force development [31, 32]. In addition, a gradual decrease in the force profile over time was recorded. Therefore, for future implementations, the stimulation protocol will need to be optimised to reduce fatigue and maintain a rhythmic tetanic contraction, as constant as possible, during the 2D flow MRI. This can be achieved by adapting parameters of the NMES protocol, such as increasing the pulse width to 1000 µs [33] or reducing the frequency to 50 Hz [34].

## Conclusion

This paper presents an open-source, low-cost grip force sensor designed for use in an MR environment. The sensor only reduced the SNR by 3.7% and induced subtle RF artefacts even when placed inside the coil. Furthermore, the sensor maintained full functionality within the MR environment. Some challenges were observed, such as the recording of negative force values caused by excessive movement of the sensor during scanning and a grip force offset correlated with RF power, which was shown to be resolved by adequate cable management. As these limitations are manageable if careful attention is paid to cable management and proper wrist immobilisation, we conclude that the sensor demonstrates strong MRI compatibility, with negligible influence on the MR signal and minimal impact of MR acquisition on device performance.

In this work, the grip force sensor was evaluated for its feasibility in dynamic muscle MRI to characterise forearm muscle activity in healthy subjects. However, its applications can extend to scenarios where grip force can be recorded in an MR scanner, such as fMRI, MR spectroscopy during exercise, or in patient populations with pathologically affected forearm muscles, as seen in myotonic dystrophy. In conclusion, the proposed low-cost, open-source grip force sensor demonstrated MRI compatibility with minimal signal interference and accurate force measurement; however, further validation is needed for broader application across different field strengths and scanner configurations.

## Supplementary Information

Below is the link to the electronic supplementary material.

Supplementary file1 (DOCX 649 kb)

## Data Availability

Schematics, building instructions, data and code to reproduce the figures of this manuscript are available at https://github.com/BAMMri/Open-Grip-Force.
