# Supplementary material for: Open-source, MRI-compatible grip force sensor for dynamic muscle imaging
Source: MAGMA. 2025 Jul 25;38(4):717–25. doi: 10.1007/s10334-025-01282-y (PMC12443942; doi:10.1007/s10334-025-01282-y)
Supplement: Supplementary file 1 — Supplementary file1 (DOCX 649 kb) [file 10334_2025_1282_MOESM1_ESM.docx]

Supplementary Figures


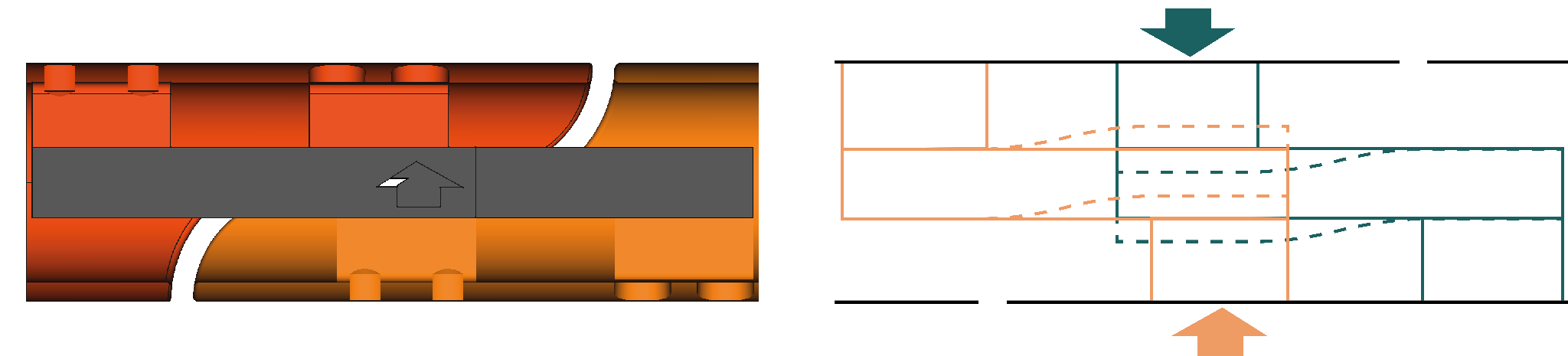


Figure S1: Cut through view of the force sensor with corresponding schematic, over-simplified, and exaggerated compression load deformation (dotted lines) of the beam load cells during grip application. The two load beam cells are represented with different colours. This diagram is a simplified representation, provided for conceptual clarity only and intended to support qualitative understanding of the load application; it is not drawn to scale and does not reflect detailed load analysis.


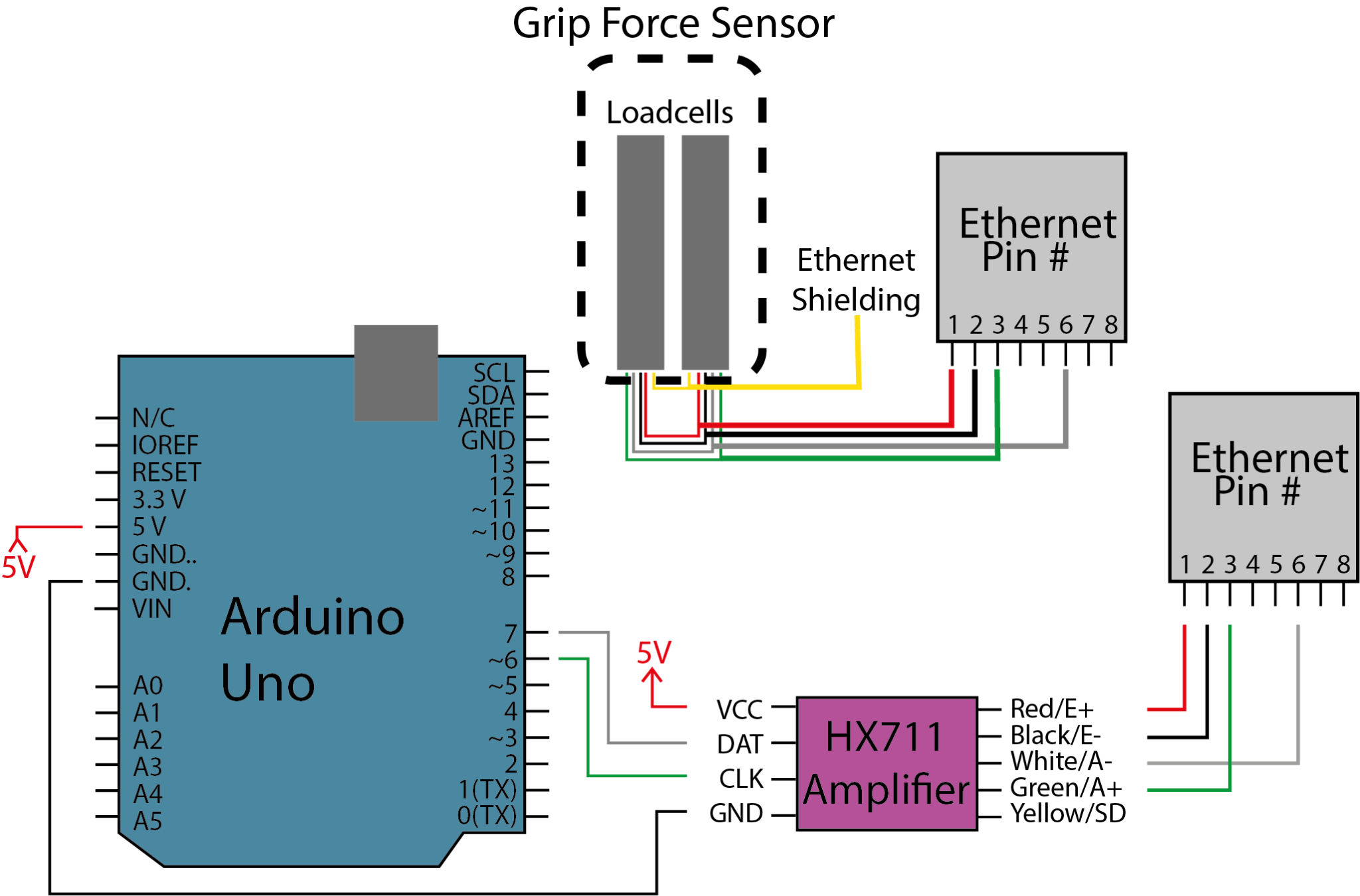


Figure S2: Schematic sketch of the electronic components and wiring utilised for the grip force sensor (without NMES addition). For details about the NMES extension, please review the building instruction manual linked in the GitHub repository [https://github.com/BAMMri/Open-Grip-Force](https://github.com/BAMMri/Open-Grip-Force.git).


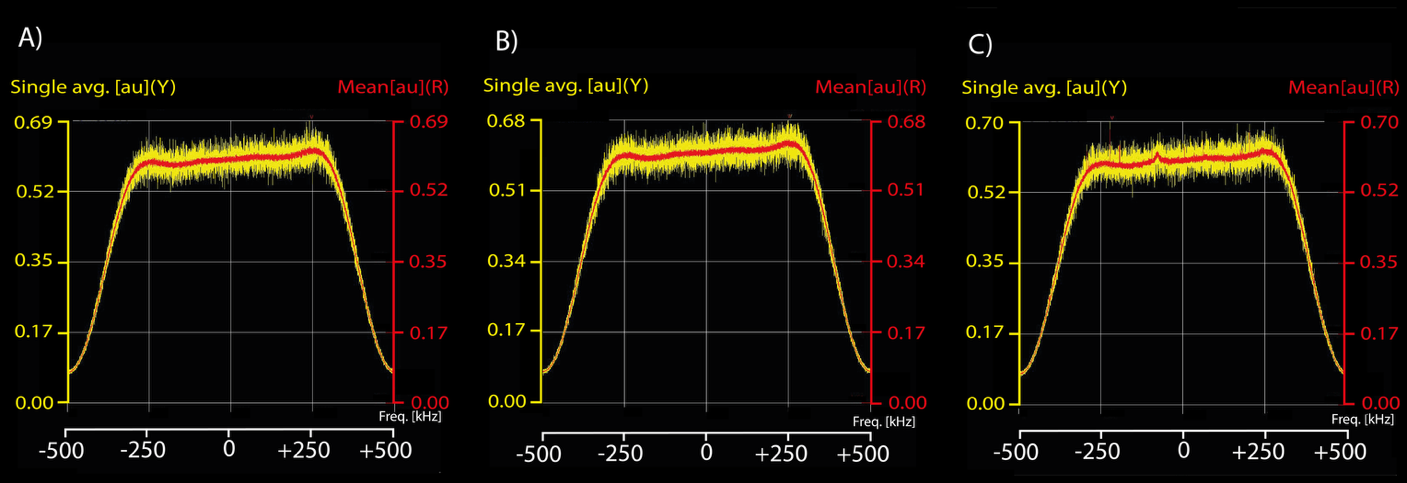


Figure S3: RF noise spectra acquired using the Siemens RF noise service sequence with an 18-channel flex coil and a cylindrical water phantom under different conditions. (A) No additional equipment is installed in the scanner room. (B) The full grip-force setup is installed as described in the methods. (C) The force sensor is placed next to the water phantom inside the scanner. The yellow line is the raw measured signal, and the red line is the smoothed signal, as provided by the scanner manufacturer. No qualitative difference is observed between scenarios A and B, as mean and maximum of the smoothed RF noise remain nearly constant. Only in scenario C does a peak appear in the noise spectrum around -65 Hz from the proton resonance frequency, while mean and maximum smoothed RF noise remain comparable in magnitude to scenarios A and B.
